# Supplementary material for: MetaQTL: a package of new computational methods for the meta-analysis of QTL mapping experiments
Source: BMC Bioinformatics. 2007 Feb 8;8:49. doi: 10.1186/1471-2105-8-49 (PMC1808479; doi:10.1186/1471-2105-8-49)
Supplement: Additional File 7 — MetaQTL Package : jar file and tutorial. This Zip archive contains both the MetaQTL JAR file and the files of the tutorial. [file 1471-2105-8-49-S7.zip › org.inra.metaqtl/doc/org/thalia/bio/util/ResourceBioEntityQuery.html]

ResourceBioEntityQuery


|  |  |  |
| --- | --- | --- |
| **Overview** \| **Package** \| **Class** \| **Use** \| **Tree** \| **Deprecated** \| **Index** \| **Help** | |  |
| **PREV CLASS**   **NEXT CLASS** | **FRAMES**    **NO FRAMES**     **All Classes** |  |
| SUMMARY: NESTED \| FIELD \| CONSTR \| METHOD | DETAIL: FIELD \| CONSTR \| METHOD |  |


---


## org.thalia.bio.util Class ResourceBioEntityQuery

```
java.lang.Object
  ResourceQuery
      org.thalia.bio.util.ResourceBioEntityQuery
```

---

``` public class ResourceBioEntityQuery extends ResourceQuery ```

This class makes it possible to make request
on resource content using an array of requested
entities.

**Author:**
:   Jean-Baptiste Veyrieras

---

**Constructor Summary**

`ResourceBioEntityQuery()`


| **Method Summary** | |
| --- | --- |
| `static IResource[]` | `getResources(IResource resource, IBioEntity[] entities)` |
| `static boolean` | `isAssignableEntities(IResource resource, IBioEntity[] entities)`             Returns true if all elements of entities are included in the resource. |
| `static boolean` | `isAssignableEntity(IResource resource, IBioEntity entity)`             Returns true if the resource contains the given entity. |

**Methods inherited from class java.lang.Object**

`equals, getClass, hashCode, notify, notifyAll, toString, wait, wait, wait`

**Constructor Detail**

### ResourceBioEntityQuery

```
public ResourceBioEntityQuery()
```


**Method Detail**

### getResources

```
public static IResource[] getResources(IResource resource,
                                       IBioEntity[] entities)
```

---


### isAssignableEntities

```
public static boolean isAssignableEntities(IResource resource,
                                           IBioEntity[] entities)
```

:   Returns true if all elements of entities are included in the
    resource.

    :   **Parameters:**: `resource` -: `entities` - **Returns:**: **See Also:**: `isAssignableEntity()`

---


### isAssignableEntity

```
public static boolean isAssignableEntity(IResource resource,
                                         IBioEntity entity)
```

:   Returns true if the resource contains the given entity.

    :   **Parameters:**: `resource` -: `entity` - **Returns:**


---


|  |  |  |
| --- | --- | --- |
| **Overview** \| **Package** \| **Class** \| **Use** \| **Tree** \| **Deprecated** \| **Index** \| **Help** | |  |
| **PREV CLASS**   **NEXT CLASS** | **FRAMES**    **NO FRAMES**     **All Classes** |  |
| SUMMARY: NESTED \| FIELD \| CONSTR \| METHOD | DETAIL: FIELD \| CONSTR \| METHOD |  |


---
